# Fixations, blinks, and pupils differentially capture individual and interpersonal dynamics in role-asymmetric mutual gaze interaction

**DOI:** 10.1038/s41598-026-39411-9

**Published:** 2026-02-11

**Authors:** Mehtap Çakır, Anke Huckauf

**Affiliations:** General Psychology, Ulm University, Ulm, Germany

**Keywords:** Dual eye-tracking, Interpersonal synchronization, Blink behavior, Pupil dilation, Fixation, Dyadic gaze interaction, Neuroscience, Psychology, Psychology

## Abstract

Although eye cues have proven effective in simulated gaze contact, it remains unclear how and through which eye parameters people interpret and use such cues in real interactions. We developed a real-time dyadic paradigm that restricted interaction to the eye region and incorporated asymmetrical roles and temporally structured interaction phases. One partner (listener) experienced emotion-inducing sounds, while the other (observer), unaware of the timing or content, attempted to infer the listener’s emotions solely from eye cues. Using a multi-measure approach, we analyzed fixation, blink, and pupil parameters in 25 dyads. Results showed that the parameters were shaped primarily by role- and phase-related processing demands rather than emotional valence. Blinks indexed role-specific processing demands, adapting to attentional priorities. Interpersonal blink synchronization decreased when partners’ attentional goals diverged, underscoring its dependence on attentional coupling. Fixations reflected shared attention allocation across roles, marked by active visual exploration during mutual gaze phases. Pupil dilation signaled phase-dependent arousal and cognitive effort, particularly for observers. Together, these findings reveal differential sensitivity across eye parameters, extending from attention allocation to social cognition and interpersonal coordination, and highlight the need for multi-measure frameworks to model the eyes as an integrated system for real-time social communication.

## Introduction

Eye-tracking methodology has emerged as a powerful tool for investigating cognitive and affective processes underlying social cognition by providing objective access to millisecond changes in eye parameters. This capability is particularly valuable for understanding interpersonal communication, where much of the expression and perception of cognitive and affective information may operate through automatic, implicit channels.

The eyes occupy a central position in human social interaction due to their exceptional communicative capabilities. People prioritize attention to the eye region when viewing faces^1–3^, establishing eye contact as a fundamental mechanism for social communication. This preferential visual attention reflects the rich social information conveyed through eye-based signals. Fixation patterns serve as the primary eye-tracking parameters for understanding social attention dynamics, where fixation location indicates areas of interest and fixation duration reflects the intensity of interest^4^. When fixations are reciprocally directed toward the eyes, creating mutual eye contact, it signals shared engagement between interaction partners. The effects of direct eye contact extend beyond simple attention, as it influences how emotional expressions are evaluated^5^, signals romantic attraction^6^, enhances trust between individuals^7^, and can even facilitate altruistic behavior^8^. Remarkably, eye contact facilitates behavioral synchrony, suggesting that mutual gaze activates mechanisms for interpersonal coordination^9–11^.

**Fig. 1:**
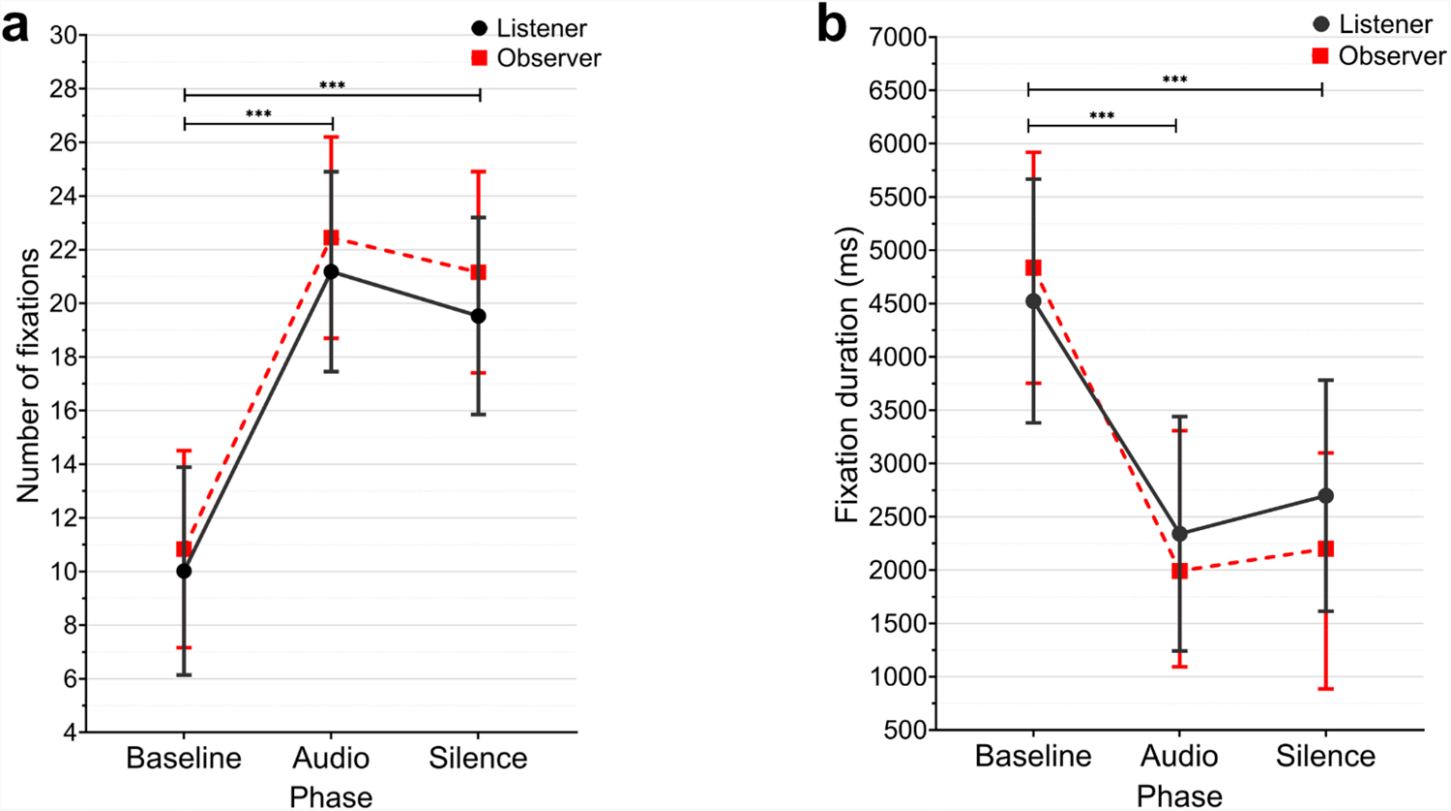
Estimated marginal means of the number of fixations (**a**) and fixation duration (**b**) by phase and role. Error bars represent 95% confidence intervals. ***p $\le$ 0.001.

**Fig. 2:**
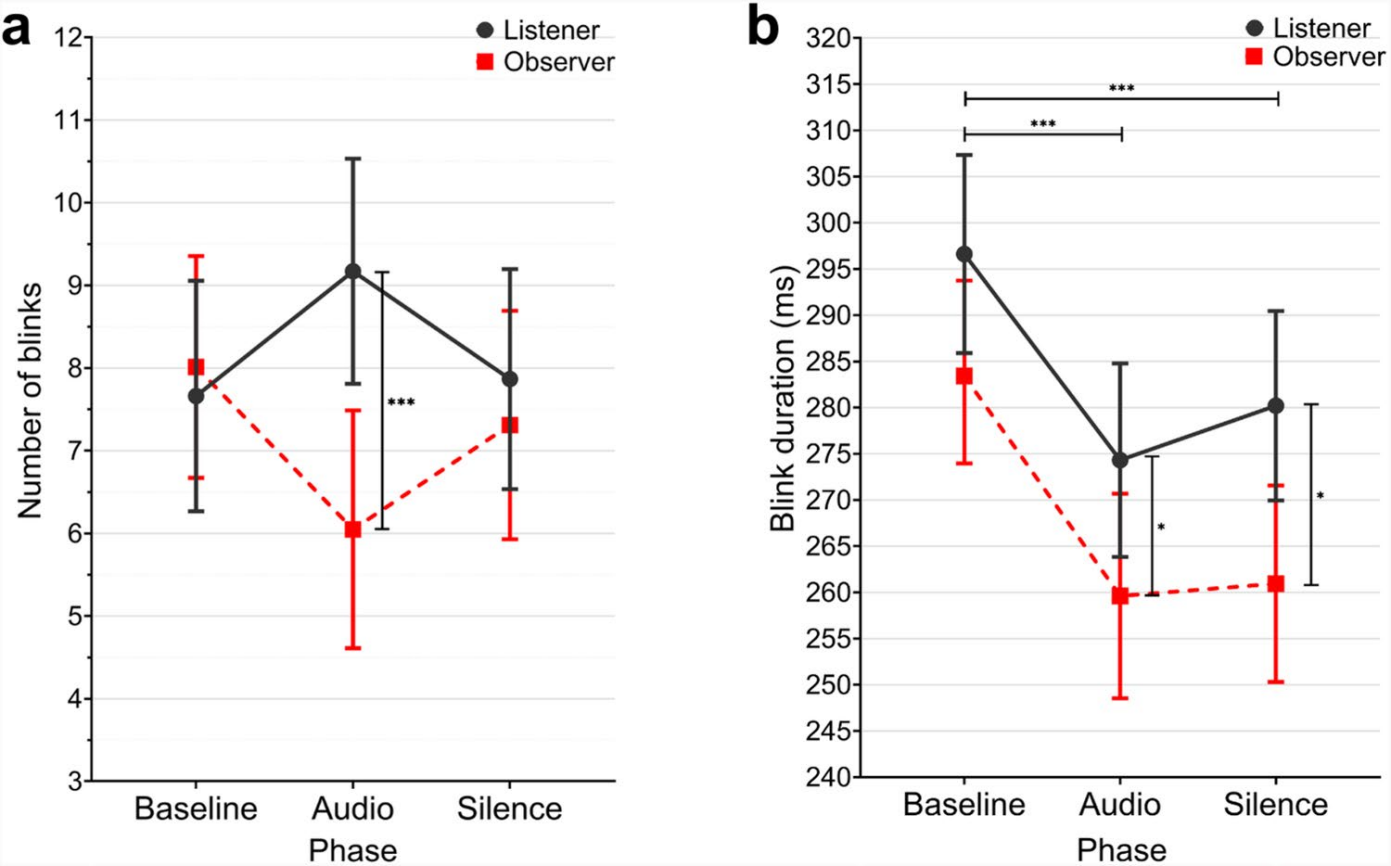
Estimated marginal means of the number of blinks (**a**) and blink duration (**b**) shown by phase and role. Error bars represent 95% confidence intervals. *p $\le$ 0.050, ***p $\le$ 0.001.

**Fig. 3:**
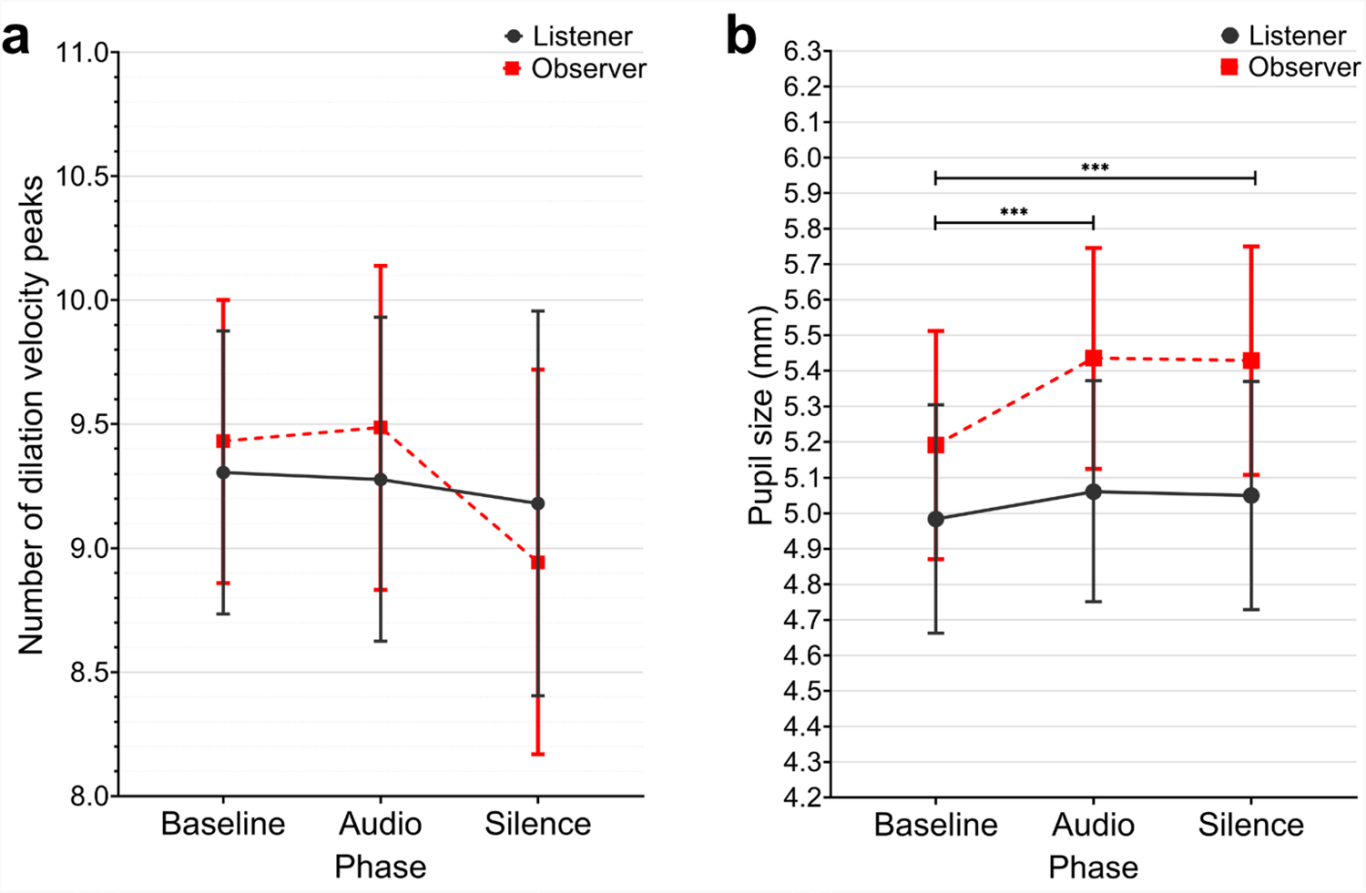
Estimated marginal means of the number of pupil dilation velocity peaks (**a**) and mean pupil size (**b**) shown by phase and role. Error bars represent 95% confidence intervals. ***p $\le$ 0.001.

**Fig. 4:**
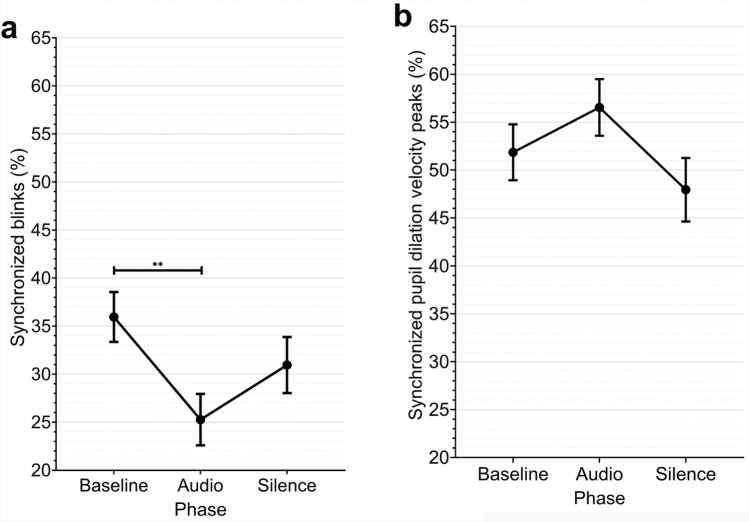
Estimated marginal means of the synchronized blinks (**a**) and synchronized pupil dilation velocity peaks (**b**) by phase. Error bars represent standard errors. **p $\le$ 0.01.

Upon investigations to understand how these communicative processes operate, two ocular measures have emerged as particularly sensitive to affective and social dynamics: blinking behavior and pupil dilation. Blinks provide information about emotional and attentional states besides their physiological role of lubricating the corneal surface. For instance, higher frequency of blinks is observed in stressful situations^12^ or when listening to sad music^13^. Blinks are reported to increase and be prolonged during internally-directed attentional tasks compared to externally-directed ones^14–16^. In social contexts, blink behavior serves as a communicative signal: during dyadic conversations, reduced blink rates and prolonged blink duration signal listener’s focused attention on the speaker’s message^17^. The communicative power of blinks is further demonstrated in a study showing that altering the blink duration of a virtual avatar affected the length of human speakers’ verbal responses^18^. Pupil dilation, controlled by the autonomic nervous system, serves as a real-time indicator of arousal^19,20^, reflecting fluctuations in emotional and mental activity^21^. The velocity of dilation reflects how rapidly the autonomic system responds to arousing stimuli. For example, sudden signals that capture attention, such as unexpected sounds, trigger rapid enlargement of the pupil that becomes more pronounced with increasing novelty and salience of the signal^22^. In the present study, we focus on dilation velocity peaks, which are the moments of the fastest increases in dilation, as fine-grained temporal markers of arousal. These peaks enable a more refined assessment beyond mean pupil size to capture the arousal shifts. Importantly, pupil responses also influence social perception: dilated pupils lead people to be rated as more attractive^23^, intelligent^24^, and trustworthy^25^. Correspondingly, observers’ pupils react systematically to social stimuli, dilating when attracted to faces^26^ and when viewing trustworthy individuals^27^.

Beyond individual responses, these eye measures exhibit interpersonal coordination that may reflect fundamental social interaction mechanisms. Of particular interest is the interpersonal synchrony of these measures: blink synchronization and pupil mimicry. Blink synchronization refers to the interpersonal coordination of blink onset timing, where individuals exhibit temporally aligned blinks. It is reported to reflect shared attention and information processing^28,29^. On the other hand, emerging evidence suggests that blink synchronization also plays a crucial role in social interaction quality. Studies have demonstrated that the degree of blink synchrony correlates with level of interest^28^, increases over the time of interaction and predicts teams’ problem-solving performance^30^. Particularly compelling is research showing that individuals with autism spectrum disorders (ASD), a heterogeneous population with varying profiles of social communication, exhibited reduced blink synchronization compared to neurotypical individuals^31^. This finding highlights blink synchronization’s role in effective social communication and suggests that disruptions in this automatic coordination may contribute to social difficulties. Recent work has begun clarifying conditions supporting blink synchronization. We previously demonstrated that direct mutual gaze enhances blink synchronization^9^. This positions blink synchronization as a marker of reciprocal attentional coupling when partners share aligned goals. However, many real-world interactions involve asymmetric roles where attentional demands diverge. Whether synchronization depends on shared attentional focus or persists despite divergent demands remains unclear. The present study addresses this gap by introducing role asymmetry.

Pupil mimicry refers to the phenomenon in which a person’s pupils change size in response to the pupil size of another individual they are observing. It appears to operate through mechanisms that are somewhat distinct from those underlying blink synchronization: The terminology used in studies to describe pupil mimicry such as “transfer of arousal”^32^ and “empathetic contagion”^33^ suggests that it reflects shared affective and social processing. Research has documented robust associations between pupil mimicry and trust^34,35^. Moreover, it occurs preferentially in non-competitive contexts and shows in-group effects^36–38^. The fundamental nature of pupil synchrony is evidenced by its early emergence: infants, as young as 4 months, exhibit pupil mimicry^39,40^, indicating its role as a pathway for social bonding.

However, significant gaps remain in our understanding of eye-based communication. The precise communicative limits and capabilities of the eye region remain poorly understood, particularly when isolated from other channels of nonverbal expression such as other facial expressions, vocal cues, and body language. Also, to fully understand how different eye parameters contribute to the extraction and utilization of gaze information, it is necessary to investigate multiple eye measures simultaneously. Although prior research showed that people can derive cognitive and affective information from eye region cues^41–43^, the majority of these studies utilized single-person, screen-based experimental paradigms that involved passive detection tasks featuring static facial images or simulated interaction partners, focusing exclusively on single eye measures.

To address these gaps, we developed a real-time dyadic paradigm that constrains the interaction to the eye region. Dyads were positioned face-to-face with a physical partition that occluded all facial and bodily information below the eye region, and wearing face masks that further restricted visible cues to the immediate eye area. Building on our previous work on blink synchronization during direct mutual gaze with symmetrical partner roles^9^, we introduced role asymmetry to our design. One partner (listener) experienced emotion-inducing audio stimuli (in neutral, negative, or positive context) via noise-canceling headphones, while the other (observer), who could neither hear the sounds nor anticipate their timing, attempted to detect the listener’s emotional states through eye cues alone. The experimental protocol involved three consecutive phases: baseline (gaze at fixation cross), audio (emotional induction: listener receives audio stimuli while observer watches sustaining mutual gaze), and silence (reflection: post-stimulus mutual gaze). This design allows for examination of how eye measures and dyadic synchrony unfold across different interactional phases, and role asymmetry that creates distinct task demands. Critically, observers remained naive about the timing and context of emotional induction, creating naturalistic uncertainty about when and what type of emotional communication was occurring. This uncertainty is crucial for understanding how eye measures perform under naturalistic conditions, where observers must continuously monitor for gaze cues without explicit temporal landmarks. As a key contribution of the study, we employed a multi-measure approach to acquire an integrated view of dyadic gaze communication. By examining fixation, blink, and pupil behavior simultaneously, we aimed to identify various aspects of dyadic gaze communication that each metric captures (i.e., role differences, task demands, interactional demands). We also examined the interpersonal synchronization of blink timing, and pupil dilation velocity peaks (fastest increases in dilation velocity) under these effects.

## Results

### Emotion induction validation and observer detection

As a manipulation check, we analyzed the valence and arousal ratings of the listeners, which were provided on a 9-point scale (1 to 9). Valence ratings yielded differences in the three emotional contexts (neutral: M = 5.2, SD = 1.0; negative: M = 3.44, SD = 1.53; positive: M = 7.0, SD = 0.86) with statistical significance (*x*^2^(2) = 46.58, *p* < .001). Listeners rated their valence in the positive trial higher than both the neutral (*p* < .001) and negative (*p* < .001). Also, the neutral was rated higher than the negative (*p* < .001). Arousal ratings also varied across contexts (neutral: M = 3.8, SD = 1.78; negative: M = 6.72, SD = 1.79; positive: M = 6.04, SD = 1.54) with statistical significance (arousal: *x*^2^(2) = 26.867, *p* < .001). The arousal in the negative trial was rated higher than both the neutral (*p* < .001) and positive (*p* = .006). Also, the positive trial was rated higher than the neutral (*p* < .001). The strong agreement (valence: Kendall’s *W* = 0.93; arousal: Kendall’s *W* = 0.537) shows that the listeners were consistent in their ratings. These results demonstrate that the experimental manipulation was effective.

Observers rated their listening partners’ valence and arousal in each trial on a 9-point scale (1 to 9). We observed differences in valence ratings among the three emotional contexts (neutral: M = 4.72, SD = 1.62; negative: M = 4.88, SD = 1.36; positive: M = 5.92, SD = 1.66) that reached statistical significance (*x*^2^(2) = 6.82, *p* = .033) with a weak agreement among the ratings (Kendall’s *W* = 0.136). Positive trials were rated higher than both the neutral and the negative; however, Conover post-hoc tests revealed only a trend toward significance (*p* = .063) after Bonferroni and Holm adjustments, despite medium effect sizes for positive-neutral and positive-negative comparisons (r_rb_ = -0.549 and r_rb_ = -0.562, respectively). Arousal ratings across the three emotional contexts (neutral: M = 4.28, SD = 1.88; negative: M = 5.08, SD = 2.02; positive: M = 6.04, SD = 1.54) did not differ significantly (*x*^2^(2) = 3.402, *p* = .182, Kendall’s *W* = 0.068).

### The number and duration of fixations

A MANOVA was conducted to examine the effects of phase (baseline, audio, silence), role (listener, observer), emotional context (neutral, negative, positive), and their interactions on the number of fixations and fixation duration. During baseline phase, fixations reflected visual attention to the fixation cross on the partition, whereas during audio and silence phases, fixations reflected visual attention to the partner’s eye region during mutual gaze. The multivariate tests revealed significant main effects for phase (Pillai’s Trace = .163, *F*(4, 798) = 17.701, *p* < .001, *n*^2^_p_ = .081), but not for role (Pillai’s Trace = .003, *F*(2, 398) = .551, *p* = .577, *n*^2^_p_ = .003) or emotional context (Pillai’s Trace = .009, *F*(4, 798) = .943, *p* = .438, *n*^2^_p_ = .005). Also, there were no significant interactions (all *p* < .05). The univariate analysis for fixation frequency revealed significant and large effect of phase (*F*(2, 399) = 36.368, *p* < .001, *n*^2^_p_ = .154). Post hoc analysis using Bonferroni adjustments showed that the number of fixations was significantly lower in the baseline phase than in the audio and the silence phases (Mean Difference = -11.37, *p* < .001; Mean Difference = -9.89, *p* < .001, respectively) (see Fig. 1a). Regarding fixation duration, the univariate tests of between-subjects effects revealed significant and large effect of phase (*F*(2, 399) = 20.627, *p* < .001, *n*^2^_p_ = .094). Post hoc analysis showed that fixation duration was significantly longer during the baseline compared to the audio and silence phases (Mean Difference = 2514.83 ms, *p* < .001; Mean Difference = 2232.69 ms, *p* < .001, respectively) (see Fig. 1b).

### The number and duration of blinks

A MANOVA was conducted to examine the effects of phase, role, emotional context, and their interactions on the two dependent variables of the number of blinks and blink duration. The MANOVA revealed significant multivariate effects for phase and role (Pillai’s Trace = .053, *F*(4, 766) = 5.168, *p* < .001, *n*^2^_p_ = .026; Pillai’s Trace = .039, *F*(2, 382) = 7.700, *p* < .001, *n*^2^_p_ = .039, respectively). There were no significant effects for emotional context or its interactions with other variables (all *p* > .05). The univariate tests of between-subjects effects revealed a marginally significant effect of role (*F*(1, 383) = 3.772, *p* = .053, *n*^2^_p_ = .010) and significant interaction between phase and role (*F*(2, 383) = 3.257, *p *= .040, *n*^2^_p_ = .017) for the number of blinks (see Fig. 2a). An independent t-test showed that listeners’ and observers’ number of blinks differed significantly only in the audio phase (*t*(142) = 3.548, *p* < .001, Cohen’s *d* = 0.591). Regarding blink duration, the univariate tests of between-subjects effects revealed significant effects of phase and role (*F*(2, 383) = 10.617, *p* < .001, *n*^2^_p_ = .053; *F*(1, 383) = 12.788, *p* < .001, *n*^2^_p_ = .032, respectively) (see Fig. 2b). Post hoc analyses using Bonferroni adjustments showed that blink duration was significantly shorter during the audio and silence phases compared to the baseline (Mean Difference = -23.057 ms, *p* < .001; Mean Difference = -19.455 ms, *p* < .001, respectively). An independent t-test showed that blink duration of listeners and observers differed significantly in the audio and silent phases (*t*(127) = 2.017, *p* = .046, Cohen’s *d* = 0.356; *t*(135) = 2.468, *p* = .015, Cohen’s *d* = 0.422, respectively).

### The number of pupil dilation velocity peaks and pupil size

We measured the mean number of dilation velocity peaks (the point of fastest pupil dilation) and mean pupil size during each phase. To examine the effects of phase, role, emotional context, and their interactions, we ran mixed-design ANOVAs. On the number of dilation velocity peaks, the analysis revealed no significant effects of phase (*F*(2, 92) = 0.812, *p* = .447, *np*^2^ = .017), role (*F*(1, 46) = 0.009, *p* = .924, *np*^2^ < .001), emotional context (*F*(2, 92) = 0.629, *p* = .535, *np*^2^ = .013), or their interactions (all *p* > .05) (see Fig. 3a). Descriptive statistics indicated that both listeners and observers exhibited a consistent frequency of rapid dilation across all phases. For listeners, the mean frequencies were 9.31 (SD = 2.27) in baseline, 9.28 (SD = 2.18) in audio, and 9.18 (SD = 2.82) in silence. For observers, the corresponding values were 9.43 (SD = 2.14), 9.49 (SD = 2.62), and 8.94 (SD = 2.79).

On the mean pupil size, the analysis revealed a significant main effect of phase with a large effect size (*F*(1.518, 69.825) = 24.832, *p* < .001, *np*^2^ = .351). Pairwise comparisons revealed that pupil size increased significantly from baseline (*EMM* = 5.088) to both audio (*EMM* = 5.249, *p* < .001) and silence (*EMM* = 5.239, *p* < .001) phases, with no difference between audio and silence (*p* = 1.000) (see Fig. 3b). The phase x role interaction (*F*(1.518, 69.825) = 7.255, *p* = .003, *np*^2^ = .136) revealed that observers showed larger pupil size increases from baseline (M = 5.19) to audio (M = 5.44) and silence (M = 5.43) phases compared to listeners, who showed smaller increases from baseline (M = 4.98) to audio (M = 5.06) and silence (M = 5.05). No main effect of emotion was found (*F*(2, 92) = 0.531, *p* = .590).

**Table 1:** Effect sizes (partial eta squared) for all dependent and independent variables. Only statistically significant interactions are reported in the table. **Bold** represents large and medium-to-large effect sizes following Cohen’s conventions^44^. Asterisks represent *p* values: *p $\le$ 0.050, ***p $\le$ 0.001.

Measure	Role	Phase	Emotional context	Interaction
Number of fixations	.003	.154***	.005	–
Fixation duration	.001	.094***	.003	–
Number of blinks	.010***	.000	.000	.017* (phase x role)
Blink duration	.032***	.053***	.008	–
Number of dilation peaks	.001	.017	.013	–
Pupil size	.044	.351***	.011	.136*** (phase x role)
Blink synchronization		.070***	.002	–
Dilation peak synchronization		.023	.016	–

### Interpersonal synchronization of blinks and dilation velocity peaks

We measured the percentages of interpersonal blink synchronization and examined the effects of phase and emotional context using linear mixed-effects analysis that accounted for dyad-level random effects. The results revealed a significant main effect of phase on blink synchronization (*F*(2, 184) = 6.937, *p* = 0.001). Post-hoc comparisons showed that blink synchronization was significantly higher during baseline phases than audio phases (Estimate: 14.80, 95%CI [5.21, 24.38], *p* = 0.009) (see Figure 4a). Specifically, it was highest during baseline phases (M = 35.9%, SD = 22.3%), decreased significantly during audio phases (M = 25.2%, SD = 22.8%), and showed intermediate levels during silence phases (M = 30.9%, SD = 25.0%). The contrasts between baseline and silence phases, and between audio and silence phases, were not statistically significant. No significant effects were found for emotional context (*F*(2, 184) = 0.207, *p* = 0.813) or the phase x emotional context interaction (*F*(4, 184) = 0.291, *p* = 0.883). While the experimental manipulations alone explained only 3.7% of the variance in synchrony (marginal R^2^ = .037), variance explained by the experimental manipulations and dyad differences together was 48.3% (conditional R^2^ = .483) as visualized by the spread of individual trajectories in Supplementary Figure S1.

Next, we measured the interpersonal synchronization percentages of dilation velocity peaks and examined the effects of phase and emotional context using linear mixed-effects analysis that accounted for dyad-level random effects. The results yielded no significant main effects for phase (*F*(2, 184) = 2.139, *p* = .121) or emotional context (*F*(2, 184) = 1.455, *p* = .236) (see Fig. 4b). The interaction between phase and emotional context was also non-significant (*F*(4, 184) = 0.788, *p* = .534). The experimental manipulations alone explained only 4.2% of the variance in synchrony (marginal R^2^ = .042) , while dyad differences and experimental manipulations together accounted for 11.9% of the variance (conditional R^2^ = .119).

Exploratory correlational analyses examined whether blink and pupil peak synchronization were related. The analyses revealed no significant relationships between blink and pupil synchronization in any phase (Baseline: $\rho = -0.132$, p = .54; Audio: $\rho$ = 0.093, p = .66; Silence: $\rho$ = 0.217, p = .31; see Supplementary Figure S2). With N = 24 dyads, only large correlations ($\rho$ $\ge$ .55) were detectable with adequate power. These null findings tentatively suggest distinct synchronization mechanisms for blinks and pupils, requiring replication in larger samples.

## Discussion

The present study provides a comprehensive and integrated examination of how multiple eye measures operate during dyadic interaction. By constraining the emotional expression and perception to the eye region alone, our findings revealed differential roles of fixation, blink, and pupil parameters that extends from basic attention allocation, to social cognition, and to interpersonal coordination.

Our study effectively induced listeners’ valence and arousal through emotion-inducing sounds, providing a robust foundation for examining physiological and behavioral responses. Although listeners’ self-reported valence and arousal aligned with intended emotional responses, observers’ ability to distinguish between the emotional states experienced by listeners was substantially less pronounced. Observers detected differences in valence, though with minimal consistency, whereas arousal variations went undetected. These findings suggest that the discrepancy between self-perceived and externally observed emotional states might highlight the complexity of accurately differentiating and interpreting the emotions of others solely on gaze cues^45–47^. However, eye movements revealed a more nuanced narrative that extends beyond simple emotional detection. Our multi-measure approach demonstrated that each eye parameter investigated reveals distinct information channels by capturing fundamentally different aspects of dyadic interaction such as asymmetrical social roles, task demands, and interaction phases.

First, none of the investigated eye parameters was sensitive to emotional context (see Table 1). The observation that the results did not distinguish between neutral, negative, and positive emotional contexts aligns with prior work showing that although fixations to the eye region can help detect certain emotions and instruction to search for a specific emotion (happiness, fear, disgust, or surprise) did not have a strong effect on fixation patterns^48^. Furthermore, pupil dilation reflects more than just perceived emotional valence, but is shaped by additional cognitive factors^49^, and greater dilation responses occur for highly arousing stimuli regardless of whether they are pleasant or unpleasant^19^. Similarly, during narrative immersion, blink count was reported to decrease in highly engaging parts regardless of whether the content was horror (negative) or neutral, suggesting that blink count tracks cognitive engagement more than valence^50^. In the context of our dyadic study, the findings could indicate that eye parameters appear to be driven by role-related differences and the cognitive dynamics of the interaction rather than being emotion-specific valence responses. However, an important limitation concerns measurement reliability for emotion effects. With only one trial per emotional context and 48 participants, participant-level analyses had 80% power to detect effects of f $\ge$ 0.25 (partial *n*^2^ $\ge$ .06), while dyad-level synchronization analyses (N = 24) had 80% power only for large effects (f $\ge$ 0.40). The absence of significant emotional context effects suggests such effects, if present, are small for participant-level measures and may have gone undetected in the limits of the present design. Distinguishing between true null effects and undetected small effects requires higher-powered replication.

In our design, the role asymmetry between listeners and observers created distinct task-driven processing demands, and blinks emerged as the eye parameter most sensitive to this asymmetry, though with small and small-to-medium effect sizes (see Table 1). Listeners, who were explicitly instructed to attend to the sounds and evaluate their own emotional responses, engaged in internally oriented processing. By contrast, observers’ task was externally oriented, as they were asked to monitor their partners’ eyes in order to infer their emotional state. This role asymmetry was reflected in their blinking patterns: Listeners exhibited more frequent and longer blinks than observers. Such findings align with prior work showing that goal-directed internal attentional focus is associated with increased blink frequency and duration^14–16^. Conversely, observers’ significantly fewer and shorter blinks are consistent with evidence that individuals suppress or inhibit blinking when they anticipate relevant information and aim to maintain uninterrupted visual access, thereby reducing the risk of missing critical cues in their environment^29,51–53^. For observers, minimizing blinks likely served to optimize continuous visual monitoring of their partners’ eyes, enabling them to extract subtle affective signals. Crucially, the role-driven differences in blink behavior were most pronounced during the audio phase, when emotional communication was actively unfolding and task demands were maximally divergent. This finding underscores that blinking behavior flexibly responds to social roles, attentional priorities, and the informational demands of ongoing interaction.

In addition to blink measures, mean pupil size emerged as another parameter that exhibited role effects, specifically in interaction with phase, with a large effect size (see Table 1). The observed phase $\times$ role interaction showed that observers displayed larger pupil dilation from baseline compared to listeners in audio and silence phases. The role of pupil size as a marker of autonomic arousal and cognitive effort is established in literature^19,54,55^. In a study in which participants were asked to discriminate individual emotional states of non-linguistic sounds, pupil size was reported to increase during emotion recognition and track decision-making, linking pupil responses to the the cognitive processes involved in interpreting emotional stimuli^49^. Likewise, in our study, the observed interaction could suggest that the externally oriented task of monitoring the gaze of a partner and inferring emotional states imposed greater cognitive demands than the internally focused task of experiencing and evaluating emotional sounds. Conjointly, these findings highlight blinks and pupil size as sensitive behavioral markers of role-specific processing demands in real-time social communication.

The phase manipulation in our design structured the interaction into distinct intervals, each imposing systematically varying attentional and interactional demands. Our findings revealed that multiple eye parameters were sensitive to these phase-driven effects. Specifically, systematic differences emerged across fixation patterns, blink duration, pupil size, and blink synchronization. To start with, fixation patterns provided the most direct evidence of attentional allocation, with large and medium-to-large phase-driven effects (see Table 1). The dramatic, role-independent shift from fewer, longer fixations during baseline to numerous brief fixations during mutual gaze phases underscores how different social communication contexts fundamentally reorganize visual attention strategies. This shift suggests that face-to-face interaction promotes active visual scanning behavior as individuals explore socially relevant facial features (for review, see^56^). The absence of role differences further implies that mutual gaze establishes a shared attentional framework for facial information processing.

Blink duration and pupil size findings complemented and extended this picture by revealing adaptive strategies for managing visual access and cognitive demands. During mutual gaze phases, participants shortened their blinks, a strategy that supports sustained attentional focus and reduces the likelihood of missing socially relevant visual information^29,51–53^. In parallel, pupil size exhibited the largest phase effect among all parameters (see Table 1), with dilation increasing significantly from baseline to both audio and silence phases. Given the established role of pupil dilation as a marker of autonomic arousal and cognitive effort^19,54,55^, this suggests that mutual gaze phases were experienced as more arousing and cognitively demanding, especially for observers, as discussed above. Overall, our results suggest that while mutual gaze generates a shared attentional framework for visual monitoring, role-specific task demands shape the degree to which individuals adapt their blink and pupil responses. Fixations capture the allocation of attention, whereas blinks and pupils reveal how individuals flexibly regulate visual access and effort to meet the demands of real-time social interaction.

Blink synchronization showed large phase-driven effects, with significantly lower synchrony in the audio phase compared to baseline (see Table 1). Although the effect of asymmetrical roles could not be tested directly because synchrony was computed at the dyadic level, it is implicitly embedded in the measure. Our earlier work^9^ demonstrated enhanced blink synchronization when both partners engaged in symmetric mutual gaze with aligned goals. Here, a significant reduction in synchrony occurred only during the audio phase, when the partners’ attentional goals diverged the most. Such divergent attentional demands appear to disrupt the temporal alignment of blinks between partners, indicating that blink synchronization depends on attentional coupling. The specificity of the effect (i.e., lowest synchronization when goals decoupled, partial recovery when partners could couple goals) demonstrates sensitivity to attentional alignment, converging with previous work^28–30^. Together, these findings reveal that synchronization depends on attentional coupling, not merely visual co-presence. Critically, this reduction represents a principled consequence of decoupled attention, not a “failure”.

Furthermore, the large discrepancy between marginal and conditional R² values (.037 and .483, respectively) indicates that blink synchrony varied substantially across dyads as visualized by the spread of individual trajectories in Supplementary Figure S1: Some pairs exhibited strong temporal coupling, while others showed minimal alignment in their blinks. This substantial dyad-level variability points to the influence of interpersonal factors. Because the present study included only familiar dyads, this variability may partially reflect relationship-specific factors rather than purely universal mechanisms. Consequently, although the observed phase effect appears robust, the generalizability to unfamiliar dyads remains an open question. Future research should therefore systematically compare familiar and unfamiliar dyads to examine how familiarity affects blink synchronization and whether blink synchronization also indexes interpersonal tendencies that shape how partners attune to one another.

Interestingly, while blink synchronization decreased significantly from baseline to audio, the synchrony of pupil dilation velocity peaks showed a descriptive increase across the same transition, although it did not reach statistical significance. This divergence suggests that the two synchrony measures capture distinct layers of interpersonal alignment, a hypothesis tentatively supported by exploratory analyses showing no correlation between blink and pupil synchronization across phases (see Results and Supplementary Figure S2), though limited power requires cautious interpretation. Whereas blink synchronization appears to rely on attentional coupling and thus diminishes when partners’ attentional goals diverge, pupil synchronization may reflect an affective coupling mechanism^33^ even in the absence of attentional coupling.

The significance of these findings is amplified by the experimental constraints under which they emerged. Observers operated under uncertainty: they had no access to the auditory stimuli, remained completely naive about the timing, duration, and transitions between experimental phases, and were required to monitor their partners continuously without any explicit temporal landmarks or contextual cues. Likewise, listeners were not instructed to communicate or enhance their emotional expressions to aid observers; they simply experienced the sounds. This created a naturalistic scenario where observers had to rely entirely on subtle, moment-to-moment changes in their partners’ eye behavior to infer emotional states. Given this unpredictability and the absence of explicit landmarks, our findings collectively carry profound implications for our understanding of the expressiveness and perception of gaze-based communication. The fact that observers not only detected changes in the eye cues of listeners but also responded with corresponding adaptations in their own eye behavior demonstrates that eyes function as a remarkably rich communication channel, capable of transmitting and receiving information about internal states even in the absence of explicit cooperation. Yet, this transmission lacked valence specificity. Whether explicit cooperation would alter these patterns remains an open question. Our paradigm could be adapted to vary explicit cooperation intent to enhance emotional expressions and enable systematic comparisons between spontaneous, cooperative, and non-cooperative conditions for future investigation.

Herein, a limitation should be acknowledged that provides directions methodological refinement for future research. Our baseline measurement approach could be strengthened. The current single baseline phase may not adequately capture the natural variability in eye behavior that occurs during dyadic contact. Adding an additional silence phase immediately before the audio phase would create a more valid comparison condition. Furthermore, future studies could incorporate variable phase durations to maintain more pronounced uncertainty and better simulate the unpredictable nature of real-world social interactions.

In conclusion, our study contributes to the literature by introducing a novel paradigm that isolates the eye region, incorporates asymmetrical roles, and structures real-time interactional phases, creating naturalistic uncertainty similar to real-world dyadic gaze exchanges. Using a multi-measure framework, we simultaneously measured fixations, blinks, and pupil responses to provide an integrated view of how multiple eye parameters operate in social interaction. The results showed that eye parameters exhibited differential sensitivity to role asymmetry and interaction phase. Fixation patterns primarily reflected visual information processing regardless of role, blinks were sensitive to role-specific processing demands, and pupil size tracked phase-dependent arousal and cognitive effort. Overall, our results advocate for the development of integrated, eye-based models of social communication that account for role asymmetries, interaction dynamics, and interpersonal coordination, framing the eyes as a complex system rather than isolated behavioral indicators.

## Methods

### Participants

We recruited 50 participants (32 female, $M_{age} = 26.98$, $SD_{age} = 10.9$, $Max_{age} = 62$, $Min_{age} = 19$) to form 25 dyads of familiar partners (e.g., colleagues, friends, relatives, romantic partners, acquaintances) in order to minimize confounding arousal effects in pupillometric measurements and allow more confident attribution of pupil changes to the experimental manipulation. All participants had normal or corrected-to-normal vision and participated in the study voluntarily. Participants were awarded 1.25 credit hours if they were the students of Ulm University; all others participated without compensation. The experimental procedures and the methods of data storage and use were in accordance with the Declaration of Helsinki and approved by the ethics committee board of Ulm University (263/23). Written informed consent was obtained from all participants for participation in the study. In addition, signed informed consent for publication of identifiable images was obtained from the two participants whose photographs are included in this manuscript in Figure 6b, to illustrate the experimental procedure and setting.

### Stimuli

We used the Expanded Version of the International Affective Digitized Sounds (IADS-E) database^57^ to create the emotion-inducing audio stimuli. IADS database consists of six-second-long sounds divided into ten semantic categories. Each sound is categorized based on the affective dimensions of valence, arousal, or dominance via The Self-Assessment Manikin (SAM) Scale^58^, and on three basic emotion-rating scales (happiness, sadness, and fear). To create a thirty-second-long audio clip for each trial, we assembled five sounds. For the neutral context, we selected individual sounds that have low arousal and moderate valence ratings with unidentified emotion categorization. For the positive context, we selected individual sounds that have high arousal, valence, and happiness ratings and low fear and sadness ratings. For the negative context, we selected individual sounds that have low valence and happiness ratings and high fear, sadness, and arousal ratings. All individual sounds selected had small standard deviations. For the practice trial, a shorter audio clip was created using three sounds. Those had varying levels of emotion ratings. The sounds are presented in the order assembled, with means and standard deviations, in Supplementary Table S1. Via Audacity Software, the individual sounds were edited into one audio clip for each trial, and the task instructions generated using Elevenlabs AI Voice Generator were added to the audio clips. The audio clips for the observer included brown noise, created using Audacity with an amplitude of 0.05 and sample rate of 44100 Hz, to boost noise canceling.

### Apparatus and experimental setting

We used two mobile eye-tracking glasses (Neon by Pupil Labs GmbH) to record gaze data. The two eye cameras on the glasses record gaze data with a sampling rate of 200 Hz. Neon is compliant with the General Data Protection Regulation (EU GDPR). Two wireless headphones (Momentum 4 by Sennheiser) with adaptive noise cancellation were used to present instructions and audio clips. The volume level was kept constant for all participants and trials.

The experiment took place in an experimental room without windows and with constant artificial lighting (145 lx). A white desk (size: 150 cm x 80 cm; height: 77.5 cm), a partition (size: 120 cm x 38 cm), and two identical movable, and height adjustable chairs were placed in the room. The height and position of the chairs were only adjustable with the help of the experimenter. In all trials, participants were seated at the desk, 100 cm across from each other. To isolate the role of gaze, the partition covered below the chin, and both participants wore face masks to cover below the eyes (see Fig. 6b). The partition was placed in the middle of the desk, which is 50 cm from each participant (see Fig. 6a). There was a fixation cross on both sides of the partition panel.

### Study design

**Fig. 5:**
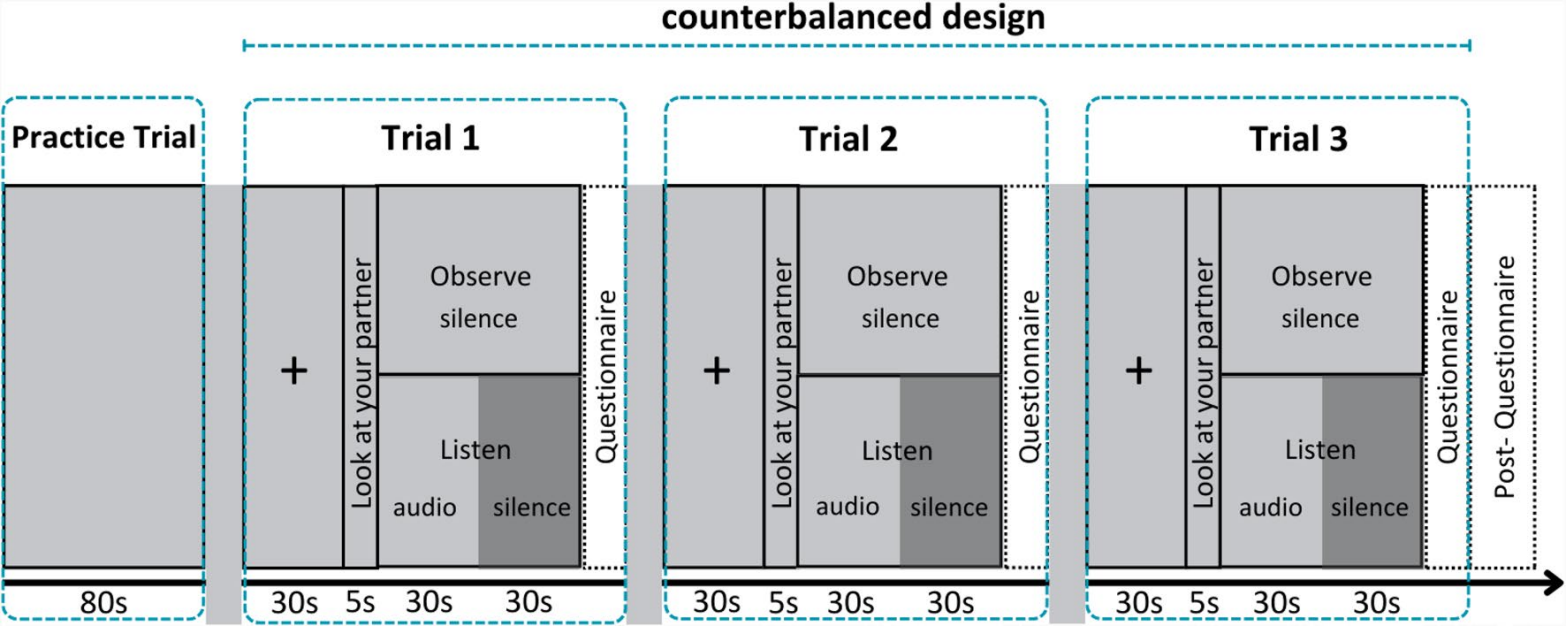
Study design. The study included three experimental trials, using a counterbalanced design. In each trial, listeners were exposed to either neutral, negative, or positive sounds. Each trial consisted of three consecutive phases. It began with the participants attending to a cross (baseline phase). Next, participants received instructions to look at their partners and either listen or observe. Listeners were exposed to a 30-second audio clip (audio phase) followed by a 30-second silence (silence phase). No additional instructions were given between the audio and the subsequent silent phase. Observers experienced 60 seconds of silence while observing their partners.

**Fig. 6:**
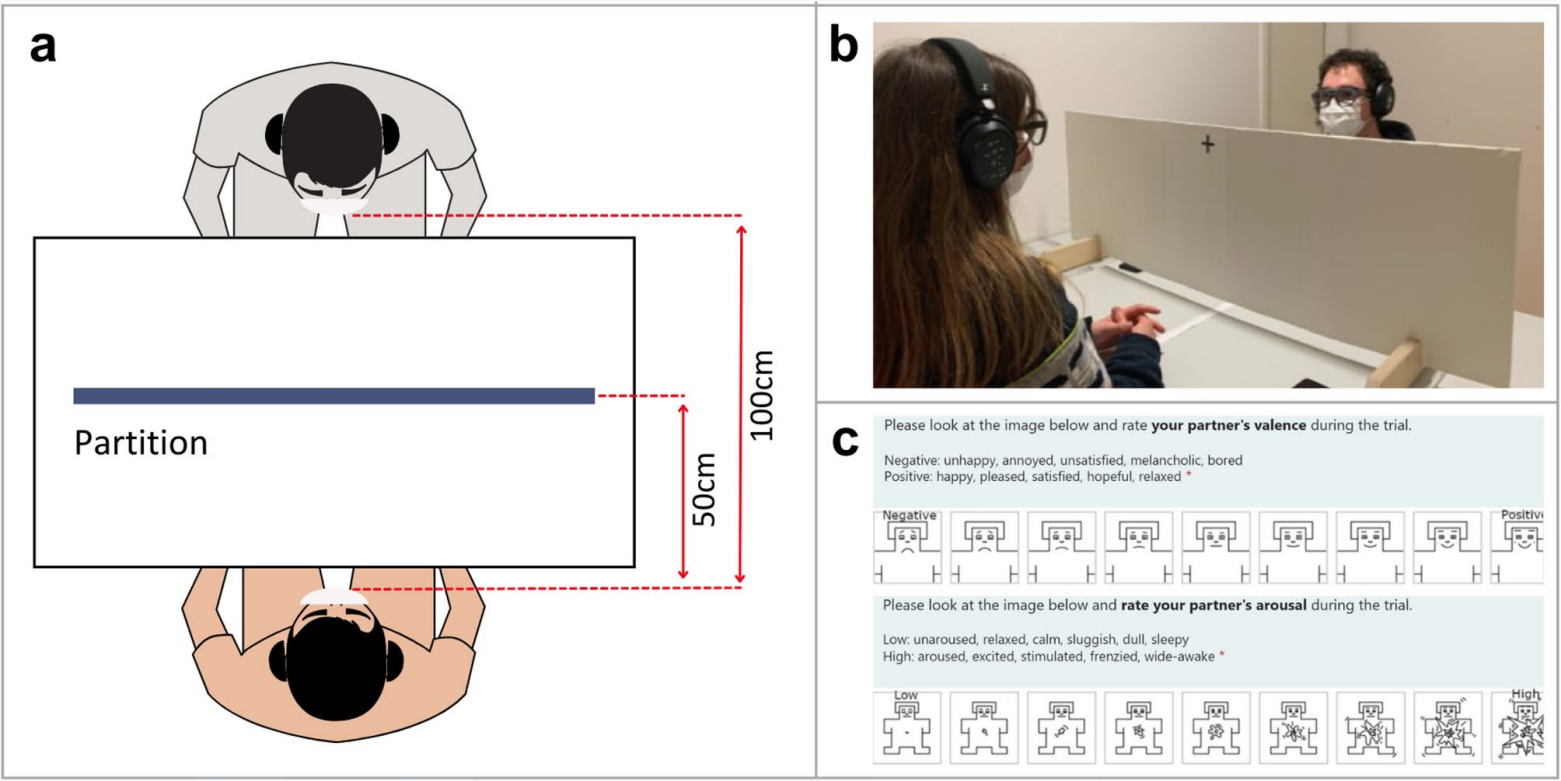
Experimental set-up and questionnaire. (**a**) Dyads sat 100 cm across each other with a partition between. (**b**) Mobile eye trackers recorded the gaze of two participants simultaneously. The partition covered the body parts below the chin, and both participants wore face masks to cover the area below the eyes. Participants looked at the fixation cross on the partition during the baseline phase. (**c**) While listeners filled out the SAM questionnaire to rate their valence and arousal in each trial, observers filled out the SAM questionnaire rating their listening partners’ valence and arousal.

Mobile eye-trackers recorded the gaze of two participants simultaneously. During the experiment, one participant was randomly assigned the role of a listener, and the other the role of an observer. We measured the eye parameters of each participant along with gathering data on perceived arousal and valence in three emotional contexts that were experimental trials: neutral, negative, and positive. In brief, the study employed a 3x3x2 factorial design with two within-subject factors (emotional context and phase) and a between-subject factor (role). Each within-subject factor had 3 levels: neutral, negative, positive for the emotional context; and baseline, audio, silence for the phase. The between-subject factor of role had 2 levels: listener and observer. The experiment was designed as a within-subjects study, and we employed counterbalanced design to vary the order of trials to avoid carry-over effects. In each trial, there were three phases of 30 seconds, which followed each other consecutively without any breaks: baseline, audio, and silence (see Fig. 5). The first phase was the baseline during which both participants attended to a cross on the partition without any audio presentation. This phase served as a control to measure baseline eye behavior. The second was the audio phase where the listener heard the instruction to look at their partner and listen, and the observer heard the instruction to look at their partner and observe. The listener listened to an emotion-inducing audio through headphones and looked at their partner. The observer observed the listener, trying to understand the listener’s emotional state, and did not hear the sounds. The third one was the silence phase where the sound stopped, the listener reflected on the induced emotion looking at the observer and the observer continued to observe their partner. Between the audio and silence phases, there was no audio instruction. Observers did not know when the audio presentation for the listener would start, and neither role knew how long the audio presentation would take.

### Procedure

First, participants were presented with written information about the experimental content and procedure. After signing the consent form, participants were familiarized with the experimental procedure. All were naïve to the purpose of the study. Then, they were randomly assigned roles and briefed about their tasks. In all trials, the experimental task for the listener was to listen to sound stimuli through the headphones and try to feel the emotion the sounds make them feel while looking at their partner. The observer’s role was to observe the listener and try to understand how the listener felt during the trial. Importantly, while listeners were aware that their partners would attempt to infer their emotional states, they received no explicit instructions to facilitate or suppress emotional expression. They were simply asked to experience the sounds authentically while maintaining mutual gaze. After the briefing, participants were taken to the experimental room and were seated at the desk. The experimenter arranged the distance between the participants. Then, participants wore face masks and eye-tracking glasses. Participants were asked to sit neutrally and avoid communicating verbally during the trials. Then, the experimenter left the room. Before the experimental trials, a practice trial was run to familiarize participants with the procedure (see Fig. 5). After the practice, three experimental trials were conducted. In each experimental trial, participants first heard an auditory signal from the headphones which counted down to the start of the trial. When participants heard the start signal, they initiated the task by first looking at the cross (baseline phase). After 30 seconds, they received the next instruction to look at their partner. Immediately following that, participants heard the instruction to either “listen” or “observe” according to their roles. After 60 s, they heard the signal to end the task and fill out questionnaires. Each trial lasted about 2 minutes. After each trial, participants filled out the SAM Scale. SAM Scale^58^ is a three-item pictographic scale that measures valence, arousal, and dominance. While the listeners filled out the questionnaire for their own experience, the observers filled out the SAM questionnaire rating their listener partners’ valence, arousal, and dominance in each trial (see Fig. 6c). After the experiment, a post-experiment questionnaire was used. A participant folder was kept concerning their experience during the experiment to ensure there were no physical conditions affecting them.

### Data preparation and analysis

Data from 50 participants (25 dyads) were used for the analysis of valence and arousal ratings. Non-parametric Friedman tests were used due to ordinal scaling to compare the main effects of emotional context. Significant effects were examined using Conover’s post hoc tests with Holm correction for multiple comparisons.

Data from 1 dyad were excluded from the eye-tracking analysis because the eye-tracker did not record the gaze data from a participant in two trials, meaning that data from 24 dyads (48 participants) were included in the fixation, blink, and pupil analyses.

For the fixation analyses, we extracted the number of fixations and mean fixation duration for each participant during each phase. All detected fixations were included without computational spatial filtering, as the experimental setup physically constrained the visible field to intended target areas. During baseline, the fixation cross on the partition served as the central visual target. During audio and silence phases (mutual gaze), the physical partition (covering below the chin) and face masks (covering features below the eyes) limited the visible field to only the partner’s eye region (see Figure 6b). Explicit instructions further directed participants to fixate the cross (baseline) or their partner (audio and silence). Manual inspection of all scene camera recordings confirmed sustained on-target gaze throughout all phases. As our experimental question concerned attention allocation during mutual gaze broadly rather than micro-level analysis of specific eye sub-regions, all fixation data were included in analyses. For the analysis of conceptually related measures of number of fixations and fixation duration, a multivariate analysis of variance (MANOVA) was employed. We examined the effects of phase, role, emotional context, and their interactions. Box’s test indicated violation of homogeneity of covariance matrices (Box’s M = 383.884, *p* < .001). Levene’s tests showed significant violations of variance homogeneity for the number of fixations based on both mean (F(17, 399) = 2.173, *p* = .005) and median (F(17, 399) = 1.860, *p* = .020). For fixation duration, violations were significant when based on mean (F(17, 399) = 2.744, *p* < .001) but not median (F(17, 399) = 1.490, *p* = .094), suggesting the presence of outliers. Therefore, we used Pillai’s Trace as our primary test statistic and applied a more conservative alpha level ($\alpha$ = .01) to reduce Type I error risk.

Another MANOVA was conducted to examine the effects of the same independent variables on the number of blinks and blink duration. Box’s test of equality of covariance matrices indicated that the assumption of equal covariance matrices was met (Box’s M = 51.044, *p* = 0.546). Levene’s test of equality of error variances suggested homogeneity of variances for both dependent variables (all *p* > 0.05). For all MANOVAs, post-hoc pairwise comparisons were performed using Bonferroni adjustments to account for multiple comparisons. All ANOVA and MANOVA analyses were conducted on SPSS Version 29.0.2.0 (20)^59^.

For the pupillometry analyses, data from the left eye were used. To prepare the pupil data for analyses, blinks were reconstructed by linearly interpolating data from 100 ms before to 150 ms after each blink. Saccades were also interpolated linearly, using a 75 ms buffer both before and after each event. Further procedures followed the guidelines proposed by Kret and Sjak-Shie (2019)^60^ when deemed necessary upon visual inspection of plots after each step: To remove dilation speed outliers, any sample with a speed exceeding the median ± 3$\times$MAD was marked as an artifact. Additionally, to minimize edge artifacts, we removed 50 ms of data preceding and following any data gap longer than 75 ms. Finally, based on visual inspection of the plots, we applied a sparsity filter: data intervals shorter than 50 ms were marked as missing if they were flanked by gaps longer than 40 ms. We calculated the missing data percentages of each experimental phase for each participant. No phase required exclusion, as none exceeded the 30% missing data threshold established for exclusion.

Firstly, we compared the number of pupil dilation velocity peak. To detect pupil dilation peaks, preprocessed pupil size data were first linearly interpolated and smoothed using a Savitzky-Golay filter (window length = 11, polynomial order = 3). We then computed the first derivative and identified local maxima in the derivative signal that exceeded 2.5 standard deviations, corresponding to rapid pupil dilation peaks. To avoid detecting multiple peaks in close proximity, we applied a non-maximal suppression threshold of 1500 ms. A 3 (emotional context: neutral, negative, positive) $\times$ 3 (phase: baseline, audio, silence) within-subjects $\times$ 2 between-subjects (role: listener, observer) mixed-design ANOVA was conducted. Mauchly’s test of sphericity showed that sphericity assumption was met for emotional context (*x*^2^(2) = 3.777, *p* = .151) and emotion x phase interaction (*x*^2^(9) = 8.923, *p* = .445), but it was violated for the main effect of phase (*x*^2^(2) = 13.340, *p* = .001). Therefore, to report phase effects and any interaction with phase, degrees of freedom were corrected using Greenhouse-Geisser correction ($\varepsilon$ = .796). Levene’s test confirmed homogeneity of variance across groups (all *p* > .05). Post-hoc pairwise comparisons of all mixed-design ANOVAs were performed using Bonferroni adjustments.

Secondly, we compared the raw mean pupil size. A 3 $\times$ 3 $\times$ 2 mixed-design ANOVA was used (as in the analysis of dilation velocity peaks). Mauchly’s test of sphericity showed that sphericity assumption was met for emotional context (*x*^2^(2) = 4.657, *p* = .097), but it was violated for the main effect of phase (*x*^2^(2) = 17.195, *p* = <.001) and emotion x phase interaction (*x*^2^(9) = 54.311, *p* = <.001). Therefore, to report phase effects and any interaction with phase, degrees of freedom were corrected using Greenhouse-Geisser correction ($\varepsilon$ = .759, and $\varepsilon$ = .654, respectively). Levene’s test confirmed homogeneity of variance across groups (all *p* > .05), supporting the validity of the mixed-design ANOVA approach.

To examine eyeblink synchronization between partners within each dyad, we adopted the methodology described by Cakir and Huckauf (2024)^9^ and Koyasu et al. (2022)^61^. Specifically, we employed a 3-second analysis window centered on each blink onset of the listener (designated as the “reference”) and assessed corresponding blink activity in the observer (designated as the “test”). This window spanned 1.5 seconds before and after the reference blink onset. For each blink of the reference, we identified the nearest blink in the test and calculated the temporal lag (i.e., asynchrony) by subtracting the reference’s blink onset timestamp from the test’s. These intersubject asynchronies, recorded within the 3-second windows, were aggregated for each dyad across all experimental phases. We then computed the proportion of synchronized blinks occurring within these windows relative to the total number of blinks in each phase, yielding a synchronization percentage for each dyad.

To investigate synchrony in pupil dilation between dyad partners, we developed a method analogous to the blink synchrony analysis by using the timestamps of detected pupil dilation peaks. Based on previous findings indicating that pupil dilation typically begins around 800 ms and reaches its peak between 2 and 2.8 s^32,37,39^, we defined a 5-second analysis window extending 2.5 s before and 2.5 s after the reference pupil peak. For each detected peak in the reference, the nearest peak in the test was identified, and the temporal lag was calculated by subtracting the reference peak timestamp from the test’s. These asynchronies were recorded for all dyads across phases. Finally, we computed the percentage of synchronized pupil dilation peaks that fell within ±2.5 seconds of each reference peak, relative to the total number of peaks in each phase.

Linear mixed-effects models were fitted using the lmer function from the lme4 package in R^62^, to examine the effects of phase and emotional context on dyadic blink and pupil dilation synchronization. The models included random intercepts for dyad pairs to account for individual differences [synchrony percentage $\sim$ Phase * Emotion + (1 | DyadID)]. Model terms were tested using F-tests with Satterthwaite approximation for degrees of freedom. Post-hoc pairwise comparisons were conducted using estimated marginal means with Tukey adjustment for multiple comparisons.

## Supplementary Information

Supplementary Information.

## Data Availability

The datasets generated and analyzed during the current study are publicly available in the Zenodo repository and can be accessed at https://doi.org/10.5281/zenodo. 17164720.
