# Supplementary material for: Fixations, blinks, and pupils differentially capture individual and interpersonal dynamics in role-asymmetric mutual gaze interaction
Source: Sci Rep. 2026 Feb 11;16:6147. doi: 10.1038/s41598-026-39411-9 (PMC12902069; doi:10.1038/s41598-026-39411-9)
Supplement: Supplementary file 1 — Supplementary Information. [file 41598_2026_39411_MOESM1_ESM.pdf]

Fixations, Blinks, and Pupils Differentially Capture Individual and Interpersonal Dynamics in Role-Asymmetric Mutual Gaze Interaction

Mehtap Çakır[1,*] and Anke Huckauf[1]

[1]General Psychology, Ulm University, Ulm, Germany

*corresponding author: M.Ç. (email: mehtap.cakir@uni-ulm.de)

Supplementary Table S1

Trial	Sound ID	Classification	AroMN	AroSD	ValMN	ValSD	DomMN	DomSD
Practice	0315	FS	6.29	1.27	4.25	1.29	5.25	2.23
Practice	0811	H	5.50	1.44	3.91	1.85	5.41	1.87
Practice	0708	U	4.50	2.11	2.68	1.78	5.50	2.54
Neutral	0692	U	4.91	1.19	4.36	0.90	5.18	1.50
Neutral	0528	U	3.68	1.55	5.14	1.08	6.18	1.74
Neutral	0318	U	4.92	1.53	4.63	1.13	5.33	1.97
Neutral	0844	U	3.76	1.64	3.88	1.27	5.44	2.43
Neutral	0829	U	3.82	1.53	5.14	0.99	6.27	1.64
Negative	0209	F	5.83	1.83	2.29	1.63	3.96	2.73
Negative	0604	F	7.32	2.15	2.52	1.39	2.64	2.31
Negative	0225	F	7.59	1.82	2.09	0.92	3.23	2.31
Negative	0291	F	7.14	1.49	2.73	0.88	3.18	1.79
Negative	0355	FS	6.50	1.79	1.82	1.30	4.18	2.77
Positive	1109	H	7.68	1.11	7.80	1.15	5.60	2.06
Positive	1114	H	7.92	1.21	7.75	1.15	6.21	1.98
Positive	1085	H	7.77	1.02	8.09	1.19	6.50	1.82
Positive	1076	H	7.92	1.35	7.63	2.04	5.83	2.22
Positive	1084	H	6.27	1.58	6.95	0.95	5.82	1.65

U = Unidentified, H = Happy, F = Fear, FS = Fear and Sad

Supplementary Table S1. The sounds in the order assembled to create the emotion-inducing audio clips, with means (MN) and standard deviations (SD) of the affective dimensions of arousal, valence, and dominance.

Supplementary Figure S1

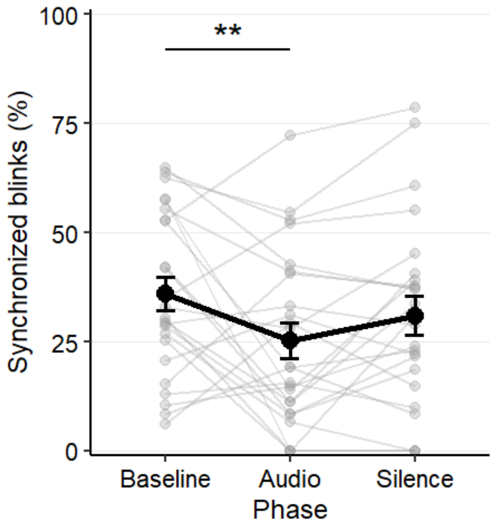

Supplementary Figure S1. Individual dyad trajectories of blink synchronization across experimental phases. Gray lines represent individual dyads (n = 24), while the bold black line shows the population mean with standard error bars. The substantial spread of individual trajectories illustrates the large between-dyad variability (conditional R^2 = .483), with some dyads maintaining consistently high synchronization across all phases while others show consistently low synchronization, despite the overall population-level decrease from baseline to audio phase (p = .003).

Supplementary Figure S2

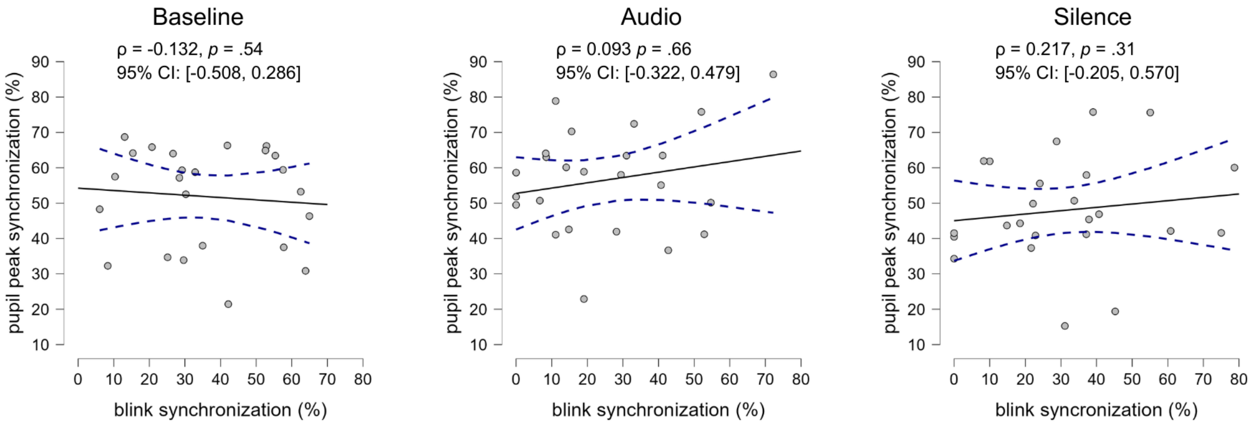

Supplementary Figure S2. Exploratory correlational analyses between blink synchronization and pupil dilation peak synchronization by phase (n = 24). Spearman correlations revealed no significant relationships in any phase. The null findings suggest blink and pupil synchronization may reflect distinct mechanisms. These exploratory findings require replication in larger samples.
